# Glycosaminoglycans and Their Mimetics

**DOI:** 10.3390/molecules22010020

**Published:** 2016-12-25

**Authors:** Vito Ferro

**Affiliations:** School of Chemistry and Molecular Biosciences, the University of Queensland, Brisbane QLD 4072, Australia; v.ferro@uq.edu.au; Tel.: +61-7-3346-9598

Glycosaminoglycans (GAGs) are linear, polyanionic polysaccharides that are ubiquitous on the mammalian cell surface and in the extracellular matrix and are generally found attached to a protein core as part of a proteoglycan. They possess enormous structural diversity and mediate a plethora of biological and pathological processes by interacting with a variety of proteins. Heparan sulfate (HS) is well known for such interactions with, for example, growth factors and cytokines, while the related GAG heparin has been in clinical use as an anticoagulant for decades. Interest continues to grow in understanding the precise nature of the interactions between GAGs and their binding partners and in defining specific biologically active sequences or arrangements of domains. Advances in these areas will advance our knowledge of disease processes and drive the discovery of new GAG mimetics as therapeutics.

In this special issue Farrugia and coworkers [1] review recent advances in the chemical synthesis of HS oligosaccharides as well as the chemical modification of various polysaccharides to produce HS mimetics. In addition, they examine chemoenzymatic approaches to HS oligosaccharides and the production of proteoglycans via recombinant techniques which are then post-translationally modified with HS chains. New advances in chemical synthesis are further illustrated by Gardner and coworkers [2] who report on the synthesis of heparin-related tetra- to octasaccharides containing orthogonal protecting groups at the O-6 position of glucosamine residues, allowing access to defined oligosaccharides with relevant sulfation patterns for future biological studies.

The low molecular weight heparins (LMWHs) are derived from the parent unfractionated heparin and are extensively used anticoagulant drugs in their own right. However, they are also mixtures and thus the characterization of chain length distribution of LMWHs is also complicated. Bisio and coworkers [3] determined the molecular weight parameters (Mw and Mn) of several LMWHs in clinical use by HP-SEC combined with a triple detector array and compared the results with those obtained using conventional detection methods. Some important differences among Mw and size distribution values were found and the results should aid the development of Mw standards for LMWHs. Staying on the theme of blood coagulation, Al-Horani and coworkers [4] screened an in-house library of sulfated GAG mimetics and identified the first small molecule allosteric inhibitors of plasmin. Plasmin is a serine protease that promotes intravascular dissolution of fibrin clots and its inhibition is clinically relevant for the management of various conditions, e.g., excessive blood loss during surgery, haemophilia etc. This work thus provides a good starting point for the future development of fibrin inhibitors with improved selectivity.

GAGs play important roles in the central nervous system (CNS) where they are crucial for brain development and homeostasis, and are involved in the pathologies of various neurological disorders. Kwok and coworkers [5] review current GAG mimetic strategies for CNS disorders and discuss some possible targets that may also be addressed using this approach. The review by Sirangelo and coworkers [6] focuses on the role of GAGs in amyloid aggregation and toxicity, which is a hallmark of neurodegenerative disorders such as Alzheimer’s disease. The GAGs present in amyloid deposits are distinct from those found in healthy tissues. Wall and coworkers [7] exploit this feature and show that heparin-reactive peptides, such as “p5+14”, can specifically target amyloid deposits. They demonstrate that radiolabeled p5+14 peptide shows selectivity for amyloid deposits over healthy tissue and thus has potential as an imaging agent for amyloid in patients. This product is currently in preclinical development. The theme of aggregation is also the subject of the contribution by Gui, Ouyang and coworkers [8]. However, in this case they show that heparin can inhibit the aggregation of nanocrystals of calcium oxalate mono- and dihydrate by increasing their aqueous stability, and thus has the potential to prevent the formation of urinary calculi (stones).

Herpes simplex viruses (HSV) are among many viruses that use HS as an entry receptor. In their review, Tiwari and coworkers [9] discuss advances in our understanding of HS-mediated HSV infection, particularly the role of the rare 3-O-sulfated glucosamine residues in this process. They also review the use of various HS mimetics to prevent HSV infection and associated tissue damage. Finally, Martel-Pelletier and coworkers [10] contribute a review on chondroitin sulfate (CS), a GAG that is used to treat osteoarthritis. They have found that there are many differences in CS composition from different sources, due to the differences in source tissue and methods of extraction and purification, which can lead to differences in biological effects. They conclude that CS product quality should be standardized and better regulated.
